# Inductively-Coupled Plasma Mass Spectrometry–Novel Insights From an Old Technology Into Stressed Red Blood Cell Physiology

**DOI:** 10.3389/fphys.2022.828087

**Published:** 2022-02-07

**Authors:** Daniel Stephenson, Travis Nemkov, Syed M. Qadri, William P. Sheffield, Angelo D’Alessandro

**Affiliations:** ^1^Department of Biochemistry and Molecular Genetics, University of Colorado Denver–Anschutz Medical Campus, Aurora, CO, United States; ^2^Faculty of Health Sciences, Ontario Tech University, Oshawa, ON, Canada; ^3^Centre for Innovation, Canadian Blood Services, Hamilton, ON, Canada; ^4^Department of Pathology and Molecular Medicine, McMaster University, Hamilton, ON, Canada

**Keywords:** inductively coupled plasma, metabolomics, iron, calcium, sodium, potassium, RBC death

## Abstract

**Background:**

Ion and metal homeostasis are critical to red blood cell physiology and Inductively Coupled Plasma (ICP) is a decades old approach to pursue elemental analysis. Recent evolution of ICP has resulted in its coupling to mass spectrometry (MS) instead of atomic absorption/emission.

**Methods:**

Here we performed Inductively-coupled plasma mass spectrometry (ICP-MS) measurements of intra- and extra-cellular Na, K, Ca, Mg, Fe, and Cu in red blood cells undergoing ionic, heat, or starvation stress. Results were correlated with Ca measurements from other common platforms (e.g., fluorescence-based approaches) and extensive measurements of red blood cell metabolism.

**Results:**

All stresses induced significant intra- and extracellular alterations of all measured elements. In particular, ionomycin treatment or hypertonic stress significantly impacted intracellular sodium and extracellular potassium and magnesium levels. Iron efflux was observed as a function of temperatures, with ionic and heat stress at 40°C causing the maximum decrease in intracellular iron pools and increases in the supernatants. Strong positive correlation was observed between calcium measurements *via* ICP-MS and fluorescence-based approaches. Correlation analyses with metabolomics data showed a strong positive association between extracellular calcium and intracellular sodium or magnesium levels and intracellular glycolysis. Extracellular potassium or iron were positively correlated with free fatty acids (especially mono-, poly-, and highly-unsaturated or odd-chain fatty acid products of lipid peroxidation). Intracellular iron was instead positively correlated with saturated fatty acids (palmitate, stearate) and negatively with methionine metabolism (methionine, S-adenosylmethionine), phosphatidylserine exposure and glycolysis.

**Conclusion:**

In the era of omics approaches, ICP-MS affords a comprehensive characterization of intracellular elements that provide direct insights on red blood cell physiology and represent meaningful covariates for data generated *via* other omics platforms such as metabolomics.

## Introduction

Red blood cells (RBCs) are the most abundant cell in the human body and play an essential role in oxygen transport and many additional functions relevant to systems physiology (Nemkov et al., 2018a). During maturation, the loss of nuclei and organelles maximizes the RBC hemoglobin content, but also results in a cell devoid of *de novo* protein synthesis capacity. In this view, ion homeostasis, membrane potential and signaling triggered by the intra- and extracellular levels of these ions are critical to RBC function in health and disease, and hold relevant implications for life-saving iatrogenic interventions such as storage in the blood bank for transfusion purposes (D’Alessandro et al., 2015; Kuhn et al., 2017). To maximize the oxygen-transport function, RBCs are loaded with (hemoglobin-bound) iron (Fe), with ∼two thirds of bodily iron accumulated in mature RBCs (Nemkov et al., 2018a). Iron homeostasis is critical to *de novo* generation of RBCs and modulation of RBC lifespans. Indeed, iron content constrains *de novo* erythropoiesis (Kautz and Nemeth, 2014). Also, RBC iron promotes Fenton and Haber Weiss redox reactions, that limit the RBC lifespan to 100–120 days as a function of oxidant stress (Kaestner and Minetti, 2017). Owing to oxidant and mechanical stress in circulation, healthy RBCs progressively become senescent prior to removal from circulation *via* erythrophagocytosis, by the reticuloendothelial system in the spleen and liver (Antonelou et al., 2010; Lutz and Bogdanova, 2013; Roussel et al., 2021). Stresses—such as ionic stress, starvation, or heat stress exacerbates RBCs aging *in vivo*, promoting the untimely clearance from the circulation through an atypical cell death process, some have referred to as eryptosis (Lang et al., 2012; Pretorius et al., 2016; Qadri et al., 2017a; Bissinger et al., 2019). Under physiological or pathological conditions, aging RBCs undergo a series of morphological (D’Alessandro et al., 2012; Roussel et al., 2021), biochemical (D’Alessandro et al., 2015) and metabolic changes (D’Alessandro et al., 2013; Jamshidi et al., 2020; Mykhailova et al., 2020), among which the most notable correlates to RBC clearance are increased intracellular calcium (Antonelou et al., 2010; Bogdanova et al., 2013), depletion of energy reservoirs (e.g., adenosine triphosphate—ATP) (van Wijk and van Solinge, 2005), disruption of ATP-dependent processes that upkeep membrane phospholipid asymmetry (Arashiki et al., 2016), causing phosphatidylserine (PS) externalization (Qadri et al., 2017a). Physiologically, extravascular hemolysis by means of RBC clearance prevents extracellular accumulation of damage-associated molecular pattern biomolecules, such as hemoglobin, heme, and iron in blood vessels—by diverting them to splenic catabolism (Antonelou et al., 2010). These processes are impaired in the context of stresses that accelerate aging of RBCs *in vivo* (e.g., sickle cell disease, thalassemia) (Buehler et al., 2021) or *in vitro* (blood storage) (D’Alessandro, 2021).

While physiological senescence of RBCs is influenced by age-related alterations (Ghashghaeinia et al., 2012), pathological stressors—such as extracellular hypertonicity (e.g., in the context of chronic kidney disease) (Bissinger et al., 2019; Xie et al., 2020), hyperthermia (Gershfeld and Murayama, 1988; Yurkovich et al., 2017), and energy starvation (van Wijk and van Solinge, 2005)—are encountered in a wide array of clinical conditions and can expedite the aging process (Lang et al., 2012; Qadri et al., 2017a). Given the availability of fluorescence-based approaches, classic literature has focused extensively on the influence of dysregulated calcium influx on the stability of cytoskeletal components, since intracellular calcium levels modulate multiple signaling cascades involving Ca-sensitive enzymes leading to cellular dysfunction and death (Lutz and Bogdanova, 2013; Qadri et al., 2017a). While it is established that these stressors elicit supraphysiologic intracellular Ca levels by enhancing membrane cation conductance, limited information is available about the impact of these stressors on the homeostasis of other ions. This gap in the literature is mostly explained by the lack of studies embracing traditional, or even modern omics approaches, while providing a detailed quantitative characterization of trace elements relevant to erythrocyte physiology, such as sodium, potassium, iron, copper, and magnesium, other than calcium. Inductively-coupled plasma mass spectrometry (ICP-MS) is a recent evolution of a decades-old analytical technique—previously coupled to atomic absorption/emission—that can be used to measure elements at trace levels in biological fluids (Wilschefski and Baxter, 2019). Since its introduction in the 1980s (Houk, 1986), ICP-MS has been established as the most reliable technique for quantifying metals, metalloids, and some non-metals in a wide range of samples. The approach is not only very simple, but also very sensitive (down to parts-per-trillion), linear over a wide working range of concentrations and suffering from limited interferences from background signal. Recent technical improvements that remove spectral interference (e.g., through the introduction of a quadrupole element) and increase robustness and reproducibility of the latest generation instrument have extended its application complex biological samples (Figueroa et al., 2016). In the last 2 years, methods have been developed that leverage ICP-MS for trace element analysis in serum and whole blood (Laur et al., 2020), but not specifically on RBCs. Given the high sensitivity of the approach, single RBC measurements of single elements (e.g., copper) have been described (Cao et al., 2020). Here we show the potential of this approach as a tool to generate novel insights in the study of RBC physiology, by performing trace element analysis to complement standard measurements of metabolism and eryptosis in the face of different stressors.

## Materials and Methods

### Red Blood Cells Treatment and Flow Cytometry Analysis

Sample collection and treatments were described in previous studies (Nemkov et al., 2020a). Briefly, RBCs were donated by five consenting healthy volunteers at the Canadian Blood Services (CBS) Network Centre for Applied Development (netCAD, Vancouver, BC, Canada; approved by CBS Research Ethics Board #2015.022). Leukocyte-filtered RBC concentrates were shipped to McMaster University, Hamilton, ON, Canada under refrigerated conditions. RBCs (10% hematocrit) were exposed to different pathophysiological conditions by incubation in different solutions at specified temperatures and time durations. To study the impact of starvation on RBC metabolism and cell death, RBCs were incubated, at 37°C for 48 h, in Ringer’s solution (containing 125 mM NaCl, 5 mM KCl, 5 mM glucose, 32 mM HEPES, 1 mM Mg_2_SO_4_, 1 mM CaCl_2_; pH 7.4) or in a modified Ringer’s solution with omission of either glucose alone or both glucose and CaCl_2_. Where indicated, RBCs were exposed to increased extracellular osmolality by incubation in hypertonic Ringer’s solution containing sucrose (550 mM) for 6 h. Elevation in intracellular Ca was stimulated by treatment of RBCs with the Caionophore ionomycin (10 μM for 1 h at 37°C; Sigma, St. Louis, MO, United States). To examine metabolic alterations in temperature-sensitive cell death, RBCs were incubated in Ringer’s solution for 12 h at 37°C (normothermia) or 40°C (hyperthermia). Baseline measurements were performed in RBCs drawn directly from blood bags stored (<5 days) under blood bank conditions. At the end of the respective treatment, RBC samples were centrifuged (272*g* at 4°C for 5 min) and the RBC pellet and the respective supernatants were transferred in separate cryotubes and snap frozen in liquid nitrogen. A small aliquot of RBCs from the treated samples was used to examine cellular markers of cell death using FACS analysis. Fluorescence intensity was determined in the FL1 channel with an excitation wavelength of 488 nm and an emission wavelength of 530 nm using EPICS XL−MCL (Beckman Coulter, Mississauga, ON, Canada) flow cytometer as described previously (Qadri et al., 2017b). To quantify the percentage of PS externalization, RBCs were stained with annexin V-FITC (1:200; ImmunoTools, Friesoythe, Germany) in Ringer’s solution containing an additional 4 mM CaCl_2_ for 15 min at 37°C. For the determination of intracellular Ca levels, RBCs were loaded with Fluo3/AM (2 μM in Ringer’s solution; EMD Millipore Corp., Billerica, MA, United States) for 15 min at 37°C. All data generated using FACS analysis were analyzed using FlowJo software (FlowJo^®^ LLC, Ashland, OR, United States). Frozen RBC samples were shipped, for metabolomics analyses, to the University of Colarado, Aurora, CO, United States on dry ice.

### Inductively-Coupled Plasma Mass Spectrometry Standards and Calibration

Prior to ICP-MS analysis, 10 μL of RBCs or supernatant were aliquoted into a 15 mL conical tube. 200 μL of 65% nitric acid and 20 ng/mL of gold were added into each sample followed by and addition of 100 μL of 30% hydrogen peroxide and brief vortexing. Samples were then incubated in an oven at 70°C for approximately 2 h. Following incubation, 2190 μL of MilliQ water was added to each tube (final nitric acid percentage of ∼5%) and all samples were vortexed briefly. All samples were then diluted 1:15 in a solution consisting of 20 ng/mL and 5% nitric acid. Final dilutions of 1:250 and 1:3750 were then analyzed *via* ICP-MS. Different dilutions were used to ensure all analytes fell within the calibration curves. All chemicals and materials used for ICP-MS analysis were obtained from Thermo Fisher and all ICP-MS calibrants and solutions were obtained from SPEX CertiPrep.

An internal standard mix of Bi, Ge, In, Li^6^, Lu, Rh, Sc, and Tb was prepared from a 100 μg/mL pre-purchased stock to a final concentration of 10 ppb. This mix was continuously flowed into the system throughout the run to account for instrument drift. Calibration curves were prepared for each analyte that was monitored during the run: 23Na, 24Mg, 39K, 44Ca, 57Fe, 63Cu. Calibration curves for Sodium, Potassium, and Calcium were 5-point curves prepared at 50, 75, 100, 500, and 1000 ppb. The calibration curves for Iron, Copper, and Magnesium were 8-point curves prepared at 1, 4, 10, 50, 75, 100, 500, and 1000 ppb. Lower limit of detection (LLOQ) for each analyte was determined based on endogenous concentrations of each analyte in the blank samples. Upper limit of detection (ULOQ) for each analyte was determined by monitoring carryover from increasing concentrations and ensuring carryover from any ULOQ did not exceed 20% of the LLOQ.

### Inductively-Coupled Plasma Mass Spectrometry Instrumentation

All samples were analyzed on a Thermo Scientific iCAP RQ ICP-MS coupled to a ESI SC-4DX FAST autosampler system utilizing a peristaltic pump. The optimization of the system was performed before the run by first calibrating the system with ICP-MS iCAP Q/Qnova Calibration Solution, Specpure. The system was subsequently tuned using a tuning solution consisting of Ba, Bi, Ce, Co, In, Li, and U at 1.00 ± 0.05 μg/L. To monitor performance while the system was running, we continually pumped internal standard mix *via* the peristaltic pump and monitored signal throughout the run. Additionally, quality controls of a known concentration of each analyte were injected at the beginning, throughout the run between samples, and at the end of the run. Acceptance criteria for all QCs was ±25% of the known concentration. Thermo Scientific Qtegra software was used for all data acquisition and analysis.

### Sample Preparation

Prior to ultra-high pressure liquid chromatography-mass spectrometry (UHPLC-MS) analysis, RBCs and supernatant isolates were placed on ice and diluted 1:10 (v/v) or 1:25 (v/v) with methanol:acetonitrile:water (5:3:2, v:v:v), respectively. Suspensions were vortexed continuously for 30 min at 4°C. Insoluble material was removed by centrifugation at 10,000 *g* for 10 min at 4°C and supernatants were isolated for metabolomics analysis by UHPLC-MS.

### Ultra-High Pressure Liquid Chromatography-Mass Spectrometry Analysis

Analyses were performed as previously described (Nemkov et al., 2017; D’Alessandro et al., 2019). Briefly, the analytical platform employs a Vanquish UHPLC system (Thermo Fisher Scientific, San Jose, CA, United States) coupled online to a Q Exactive mass spectrometer (Thermo Fisher Scientific, San Jose, CA, United States). Samples were resolved over a Kinetex C18 column, 2.1 × 150 mm, 1.7 μm particle size (Phenomenex, Torrance, CA, United States) equipped with a guard column (SecurityGuard™ Ultracartridge—UHPLC C18 for 2.1 mm ID Columns–AJO-8782–Phenomenex, Torrance, CA, United States) using an aqueous phase (A) of water and 0.1% formic acid and a mobile phase (B) of acetonitrile and 0.1% formic acid. Samples were eluted from the column using either an isocratic elution of 5% B flowed at 250 μL/min and 25°C or a gradient from 0 to 5% B over 0.5 min; 5–95% B over 0.6 min, hold at 95% B for 1.65 min; 95–5% B over 0.25 min; hold at 5% B for 2 min, flowed at 450 μL min^–1^ and 35°C. The Q Exactive mass spectrometer (Thermo Fisher Scientific, San Jose, CA, United States) was operated independently in positive or negative ion mode, scanning in Full MS mode (2 μscans) from 60 to 900 m z^–1^ at 70,000 resolution, with 4 kV spray voltage, 45 sheath gas, 15 auxiliary gas. Calibration was performed prior to analysis using the PierceTM Positive and Negative Ion Calibration Solutions (Thermo Fisher, San Diego, CA, United States). Acquired data was then converted from .raw to .mzXML file format using Mass Matrix (Cleveland, OH, United States). Samples were analyzed in randomized order with a technical mixture injected after every 15 samples to qualify instrument performance. Metabolite assignments were performed using MAVEN (Princeton, NJ, United States) (Clasquin et al., 2012).

### Statistical Analysis

Graphs, heat maps, and statistical analyses (either *T*-Test or ANOVA), metabolic pathway analysis, PLS-DA and hierarchical clustering was performed using the MetaboAnalyst 3.0 package.^1^ Hierarchical clustering analysis (HCA) was also performed through the software GENE-E (Broad Institute, Cambridge, MA, United States). XY graphs were plotted through GraphPad Prism 5.0 (GraphPad Software Inc., La Jolla, CA, United States).

## Results

### Impaired Ion Homeostasis in Response to Starvation, Ionic, or Heat Stress

**FIGURE 1:**
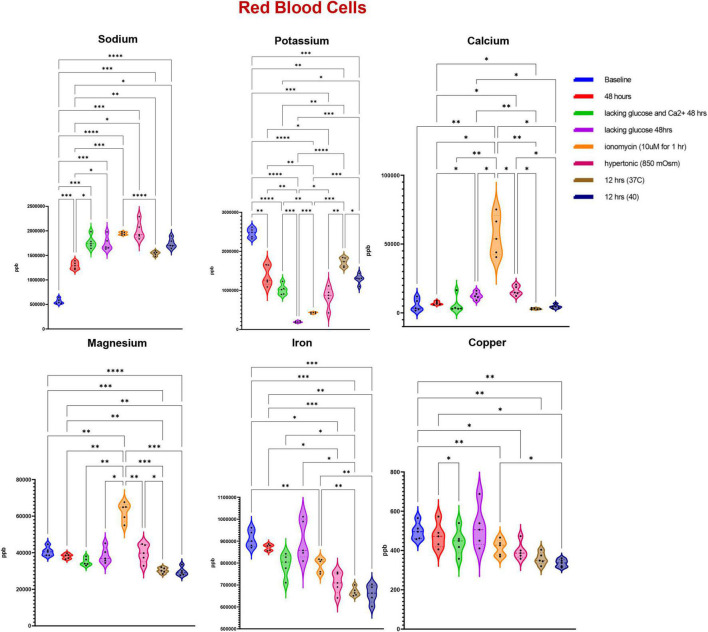
Measurements of trace elements in red blood cells exposed to starvation (glucose or glucose and calcium), ionic (ionomycin or hypertonic saline), or heat stress (40 vs. 37°C). Significance was determine *via* ANOVA with Tukey’s multiple comparison test (*p < 0.05; ^**^p < 0.01; ^***^p < 0.001; ^*⁣*⁣**^p < 0.0001).

**FIGURE 2:**
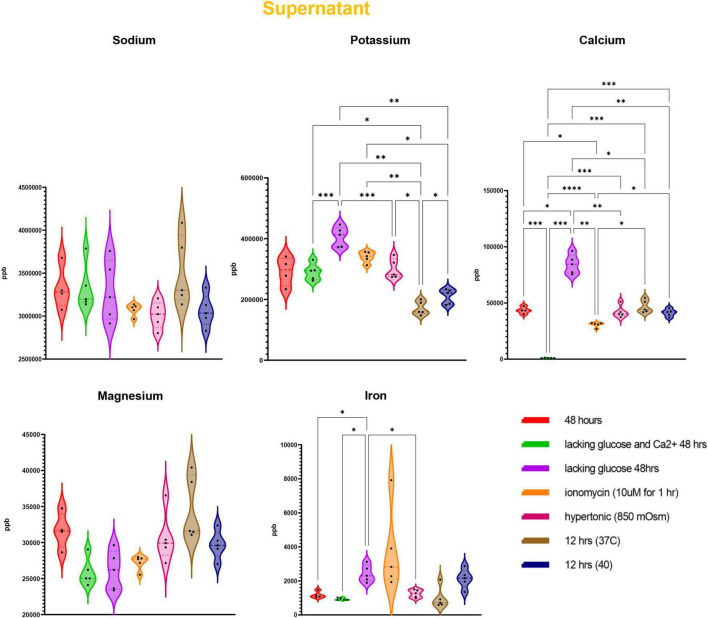
Measurements of trace elements in supernatants exposed to starvation (glucose or glucose and calcium), ionic (ionomycin or hypertonic saline), or heat stress (40 vs. 37°C). Significance was determine *via* ANOVA with Tukey’s multiple comparison test (*p < 0.05; ^**^p < 0.01; ^***^p < 0.001; ^*⁣*⁣**^p < 0.0001).

**FIGURE 3:**
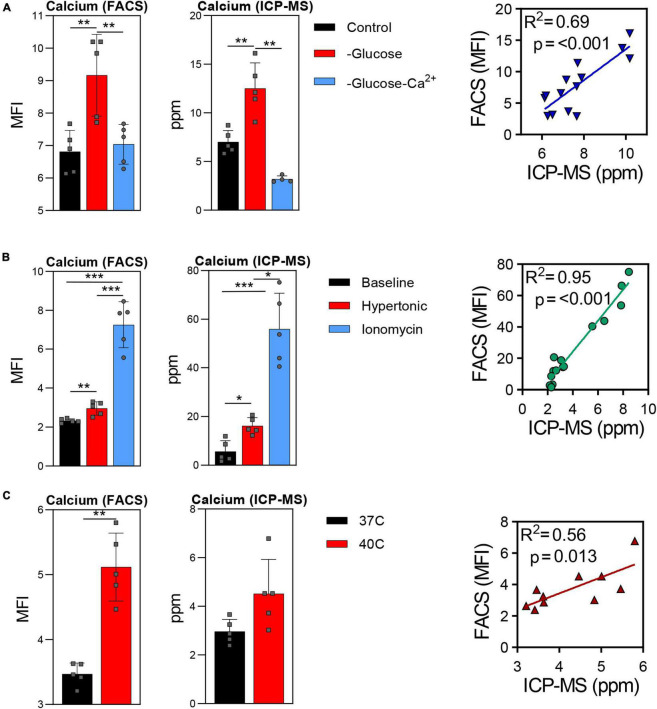
Comparison of calcium measurements *via* fluorescence-based approaches vs. ICP-MS in red blood cells after starvation **(A)**, ionic stress **(B)**, or heat stress **(C)**. *p < 0.05; **p < 0.01; ***p < 0.001.

Trace element analysis was performed *via* ICP-MS to determine the impact of starvation (glucose or glucose and Ca), ionic (10 uM ionomycin treatment or incubation in hypertonic solution—850 mOsm) or heat stress (37 vs. 40°C) on red blood cell (Figure 1) and supernatant (Figure 2) levels of sodium (Na), magnesium (Mg), potassium (K), calcium (Ca), iron (Fe), and copper (Cu). Results indicate that all stresses induce significant (*p* < 0.05) increases in intracellular levels of Na and decreases in intracellular levels of K, with highest effects observed under ionomycin and hypertonic saline interventions (Figure 1). However, lower levels of K in supernatants were observed following heat stress (Figure 2). Glucose starvation, heat stress and ion stress also depleted intracellular iron pools, with supernatant levels of iron significantly increasing in glucose-starved and ionomycin-treated RBCs (Figure 2). All stresses promoted significant decreases in intracellular levels of Cu (Figure 1). Intracellular levels of Ca spiked in response to ionomycin treatment (Figure 1), consistent with the role of ionomycin in enhancing calcium influx (Morgan and Jacob, 1994; Lang et al., 2003). Of note, significant positive correlations were observed between Ca measurements *via* ICP-MS and fluorescence-based approaches, in the context of glucose and glucose + Ca starvation (Figure 3A), ionic stress (Figure 3B), or hyperthermic stress (Figure 3C).

### Correlation Analysis of Inductively-Coupled Plasma Mass Spectrometry Calcium Data to Metabolomics Measurements

**FIGURE 4:**
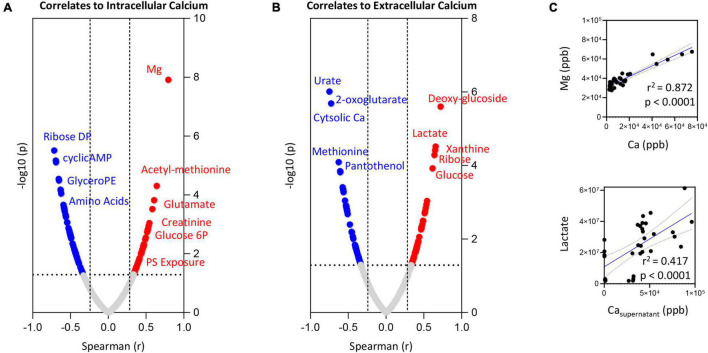
Correlation of intracellular **(A)** or extracellular **(B)** calcium levels to RBC or superantant metabolites. In panel **(C)**, highlighted correlations curves for Mg and Ca (intracellular) and Lactate and Ca (supernatant).

To expand on the observations above, we correlated trace element data (both intra- and extracellularly) with metabolomics data from RBCs exposed to starvation, ionic or hyperthermic stress, as previously described (Qadri et al., 2017a). Intra- and extracellular levels of Ca (Figures 4A,B, respectively) showed strong (significant *p* < 0.0001) positive correlations with intracellular levels of Mg and lactate, respectively (Figure 4C). Positive correlation between intracellular Ca and phosphatidylserine (PS) exposure, intracellular levels of N-acetyl-methionine, glutamate, glucose 6-phosphate and creatinine were observed (Figure 4A–full list in Supplementary Table 1). On the other hand, elevation in intracellular Ca corresponded to lower levels of pentose phosphate pathway byproducts (ribose diphosphate), cyclic-AMP, glyercophosphoethanolamine (PE), and multiple amino acids–Figure 4A. Increases in extracellular Ca corresponded elevation in intracellular markers of glycolysis (glucose, lactate) and purine metabolism (increases in xanthine and decreases in urate–Figure 4B). On the other hand, extracellular levels of Ca were negatively associated to intracellular levels of Ca, 2-oxoglutarate (also known as alpha-ketoglutarate), methionine and the CoA precursor—panthothenol (Figure 4B).

### Metabolic Correlates to Intracellular Calcium and Extracellular Potassium

**FIGURE 5:**
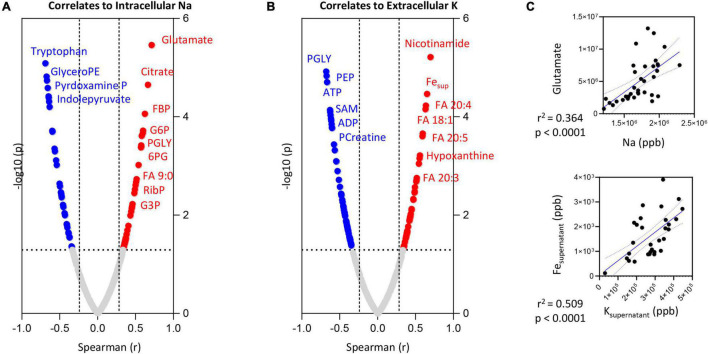
Correlation of intracellular Na **(A)** or extracellular K **(B)** levels to RBC or superantant metabolites. In panel **(C)**, highlighted correlations curves for glutamate and Na (intracellular) and Fe and K (supernatant).

Intracellular Na levels positively correlated (Spearman > 0.5; *p* < 0.0001) with intracellular levels of glutamate (Figures 5A–C), citrate and several glycolytic metabolites (glucose 6-phosphate—G6P; fructose bisphosphate—FBP; glyceraldehyde 3-phosphate—G3P; phosphoglycerate—PGLY), pentose phosphate pathway metabolites (ribose phosphate—RibP) and fatty acid oxidation products (non-anoate—FA 9:0—Figure 5A). Significant negative correlations (Spearman *r* < -0.5; *p* < 0.0001) were observed between the intracellular levels of Na and tryptophan or its indole metabolites (indolepyruvate), glyceroPE and pyridoxamine phosphate (vitamin B6 metabolism).

Extracellular K levels showed strong negative correlations (Spearman *r* < -0.75; *p* < 0.0001) with energy metabolism as inferred from the levels of adenosine triphosphate (ATP) and phosphocreatine, multiple late glycolysis metabolites (PGLY; phosphoenolpyruvate—PEP), S-adenosylmethionine (SAM—Figure 5B). Elevation in supernatant K corresponded to significant elevation in intracellular markers of purine/nucleotide metabolism (hypoxanthine, nicotinamide), several mono- (oleic acid—FA 18:1) and poly-/highly-unsaturated fatty acids (eicosa-tri- tetra- and pentaenoic acid—FA 20:3; 20:4 and 20:5—Figure 5B) as well as supernatant levels of Fe (Figures 5B,C).

### Iron Levels and Red Blood Cells Metabolism in Response to Stress

**FIGURE 6:**
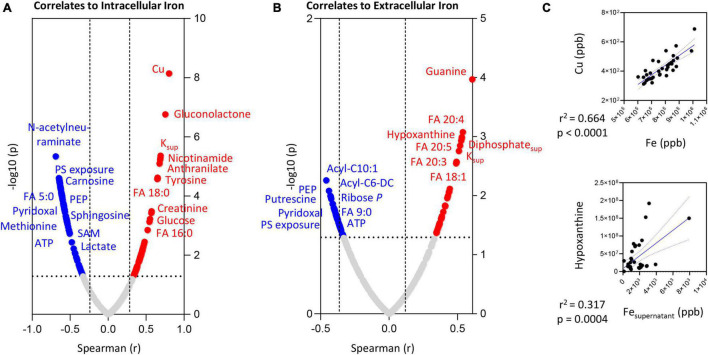
Correlation of intracellular **(A)** or extracellular **(B)** iron (Fe) levels to RBC or superantant metabolites. In panel **(C)**, highlighted correlations curves for Fe and Cu (intracellular) and Hypoxanthine and Fe (supernatant).

Intracellular and extracellular levels of iron positively correlated with the levels of saturated (palmitate—FA 16:0; stearate—FA 18:0) or mono/poly-unsaturated fatty acids (FA 18:1; 20:3; 20:4; 20:5), respectively (Figures 6A,B, respectively). Elevated supernatant levels of Fe positively correlated with hypoxanthine levels (Figure 5C).

On the other hand, both intra- and extra-cellular levels of Fe negatively correlated with PS exposure, lactate, ATP, methionine, *N*-acetylneuraminate, carnosine, pyridoxal, FA 5:0 or 9:0, medium chain acyl-carnitines (10:1 and C6-decarboxylate), and SAM (Figures 6A,B). Strong significant positive correlations (Spearman *r* > 0.8; *p* < 0.0001) between intracellular Fe and Cu were observed (Figure 6C)—and, as such, we only show the metabolic correlates for the former (given the elevated number of shared co-variates–Supplementary Table 1).

## Discussion

Given the overwhelming abundance of hemoglobin, RBCs have been historically regarded as a simple cell model to investigate mechanisms of cell death in the absence of transcriptional regulation and *de novo* protein synthesis. Recent studies have elucidated the complexity of the RBC proteome (Bryk and Wiśniewski, 2017; D’Alessandro et al., 2017) and interactome (how these proteins interact with each other) (Issaian et al., 2021). Omics studies of RBC have revealed an unanticipated complexity of this “simple” cell, highlighting a network of cytosolic enzymes with interrelated functions relevant to glycolysis, the pentose phosphate pathway and the Rapoport-Luebering shunt, i.e., the classic canonical energy and redox metabolism in the mature erythrocyte (D’Alessandro et al., 2021c). Recent studies have shown an impact of physiological aging or pathological stress [e.g., hemoglobinopathies like sickle cell disease or viral infections such as SARS-CoV-2 (Renoux et al., 2021)] on RBC metabolism, with functional implication on the RBC capacity to carry and off-load oxygen (Donovan et al., 2021), other than on the erythrocyte rheological properties that impact intra- and extravascular hemolysis (Renoux et al., 2021). A growing body of evidence has shown that mechanisms of metabolic regulation in RBC in the face of environmental changes, such as hypoxia [*in vivo* (D’Alessandro et al., 2016) or *in vitro*] (D’Alessandro et al., 2020), regulate cell-fate decisions not just in nucleated cells (Mason and Rathmell, 2011; Halama et al., 2013; Wu et al., 2017), but also in the mature RBC (Pretorius et al., 2016). Dysregulation of ion homeostasis, post-translational modifications [e.g., phosphorylation (Rinalducci et al., 2015); redox regulation (Reisz et al., 2016); methylation of isoaspartyl-damaged proteins (D’Alessandro et al., 2021b); ubiquitinylation (Tzounakas et al., 2020) or transglutaminylation (Sarang et al., 2007) of lysine residues], proteolytic cascades and vesiculation, all contribute to the regulation of RBC responses to external stimuli. Failure in metabolism negatively affect RBC survival, since energy-deficient effete RBCs are rapidly removed from the bloodstream due to impaired membrane deformability (van Wijk and van Solinge, 2005). While in cells with organelles intrinsic apoptosis cascade are mediated by nuclear and mitochondrial cascades (Arandjelovic and Ravichandran, 2015), in organelle-deficient RBCs the linkage between stress responses, ion dysregulation and apoptotic-like events remains incompletely understood.

Previous studies have detailed the impact of stress-induced calcium dysregulation in RBC physiology and metabolism (Nemkov et al., 2020a). Results showed a significant impact on the pentose phosphate pathway, glutathione and one-carbon homeostasis as a function of altered Ca-influx (Nemkov et al., 2020a), achieved either by pharmacologically intervention with drugs targeting Ca ionophores (Lang et al., 2003) or by starvation in the absence of Ca in the media. Since ionic homeostasis in RBCs is, at least in part, energy- and temperature-dependent (Gatto and Milanick, 2009; D’Alessandro et al., 2015; Yurkovich et al., 2017), similar patterns of metabolic reprogramming were observed in RBCs in response to glucose starvation or following incubation in hyperthermic conditions (40°C).

Here we expanded on the literature by measuring multiple trace elements in the context of starvation, ionic and heat stress to RBCs, by leveraging a novel technology—ICP-MS—an evolution of the original ICP coupled to atomic absorption/emission approach from a few decades ago (Wilschefski and Baxter, 2019). Of note, results obtained *via* ICP-MS for calcium showed a significant positive correlation with measurements based on classic, fluorescence-based approaches. It should be noted that, RBCs are packed with hemoglobin, therefore, fluorescent signal is usually bleached, a limitation that extends to the fluorescent-based method we used in our original study (Nemkov et al., 2020a). Other approaches, like flame photometry or ICP-MS (the method we used here) do not suffer from such limitations. In addition, the ICP-MS approach simultaneously enabled the quantitation of multiple additional elements, including Na, K, Mg, Fe, and Cu. Indeed, other than Ca influx, increases in the levels of intracellular Na and extracellular K have been previously associated with eryptosis-like cascade in RBCs under stress conditions, including but not limited to ionic, starvation or heat stress (Lang et al., 2012; Qadri et al., 2017a; Nemkov et al., 2020a). For example, failure of proton pumps at 4°C is a hallmark of the RBC storage lesion (Yoshida et al., 2019).

Of note, increases in the intracellular levels of Na and extracellular levels of K showed positive and negative correlations, respectively, to multiple glycolytic metabolites—suggestive of altered glycolysis as a function of cation homeostasis. These results are relevant yet not unexpected, since Na/K pumps in RBCs are coupled to Na/H exchangers and thus to intracellular pH, and rate-limiting enzymes of glycolysis and the Rapoport-Luebering shunt are highly pH-dependent with optimum activity in the alkaline range (Hess et al., 2000; D’Alessandro et al., 2018). Similarly, Na/K pumps play a critical role in RBC hydration and, as such, dysregulation of these systems have been proposed as markers of blood diseases (Cheng et al., 1984; Omar et al., 2017).

While dysregulation of iron metabolism has been previously associated with increased propensity for lipid stress in stored RBCs or under pathological conditions (e.g., sickle cell disease or thalassemia) (Howie et al., 2019; Buehler et al., 2021), to the best of our knowledge no study has been published correlating intra- and extra-cellular levels of iron to the intra- and extracellular RBC metabolome. In our study, iron levels correlated positively with markers of increased oxidant stress in aging RBCs—such as hypoxanthine (Nemkov et al., 2018b) and increased levels of poly-unsaturated fatty acids (consistent with activation of fatty acid desaturases) (Thomas et al., 2021). The former observation is relevant in that RBC-specific AMPD3, the rate-limiting enzyme of AMP deamination upstream to hypoxanthine synthesis—is positively regulated by intracellular calcium levels and oxidant stress (Nemkov et al., 2018b). The latter observation is interesting because it provides direct evidence of a linkage between increased phospholipase activity [by PLA2 or PLA2-like enzymes, sensitive to redox stress, like peroxiredoxin 6 (Fisher, 2018)] as a function of the levels of iron—and, predictably, reactive oxygen species. Indeed, given that the experiments were performed *in vitro* with stable media conditions, increases in the intracellular levels of free fatty acids is likely derived from lipolysis, perhaps as a function of oxidant stress. As such, it is worth noting that odd-chain short fatty acids [e.g., non-anoic acid (D’Alessandro et al., 2021a)] and acyl-carnitines [markers of membrane lipid damage repairing *via* the Lands cycle (Wu et al., 2016; Nemkov et al., 2021)] rank all amongst the top correlates to intra- and extracellular iron levels.

Our findings have potential clinically relevant translational implications. For example, recent studies have shown that storage in the blood bank promotes oxidant stress-dependent alterations of RBC physiology (D’Alessandro et al., 2012; Reisz et al., 2016), a phenomenon that in rodent models is promoted by the levels and activity of a ferrireductase STEAP3 (Howie et al., 2019) and mitigated by conditions associated with decreased RBC iron content, such as beta-thalassemia traits (Tzounakas et al., 2021) or frequent donations by recurring blood donor volunteers (Roubinian et al., 2019). Genome-wide association studies have identified glutathione peroxidase 4 (Page et al., 2021)–a mediator of ferroptosis cascade—as a potential mediator of stored RBC susceptibility to hemolysis following oxidant insults (Stolwijk et al., 2021), suggesting that blood storage events previously assimilated to eryptosis (Kriebardis et al., 2007) might indeed be explained by ferroptosis processes not just in the phagocytic cells upon extravascular hemolysis following transfusion (Youssef et al., 2018). Similar considerations could hold true in the context of increased stress (hyperthermia, oxidant stress) following viral infections, such as SARS-CoV-2 (Thomas et al., 2020).

From a biological standpoint, the study holds several limitations, among which the lack of determination of total ion contents (independently of intra- or extra-cellular compartments). Some of the eryptosis eliciting conditions may have induced hemolysis and thus interfered with the extracellular determination of some of the trace elements. Strong correlations of extracellular iron and potassium with free fatty acids and acyl-carnitines may have been influenced by vesiculation events elicited by the eryptotic treatments, suggestive that these correlations may not necessarily hold in the absence of perturbations. Our results are not conclusive with respect to the biological interpretation of some findings. For example, some experimental interventions resulted in decreases in the extracellular levels of potassium. Biological interpretations that could be speculated based on our data include either an enhancement of the Na/K ATPase (high energy consuming and thus reliant on the activation of glycolysis) or a sudden activation of the Na-K-Cl cotransporter (Lauf et al., 2006; Radosinska and Vrbjar, 2016). It cannot be ruled out that the dysregulation of calcium fluxes through ionomycin may also alter the intra-/extra-cellular levels of other divalent ions, as a result of lack of specificity or potential interactions between transport systems. As limited evidence is available supporting the presence of active iron exporters in erythrocytes (Rouault, 2019), hemolysis could be a viable interpretation for the detected levels of extracellular iron. However, low levels were detected in the supernatants of baseline, untreated cells with no detectable hemolysis. As such, an alternative explanation could be attributable to vesiculation events, with the detected iron being present in the vesicles in the supernatants that are not sorted through the gentle centrifugation step used here to separate cells from supernatants (ultracentrifugation is needed to pellet them). Indeed, RBCs shed 1 microvesicle/hour (Willekens et al., 2008) in the absence of perturbation, a phenomenon that is accelerated in the face of stress.

In summary, we provide the first ICP-MS analysis of RBCs exposed to multiple stressors, including ionic, heat or starvation stress. We thus provide the first correlation analysis of ICP-MS findings with metabolomics data. This workflow could represent a novel tool in the toolbox of investigators approaching RBC biology in health and disease. In the era of omics technologies, ICP-MS applications to trace element analysis of the atoms described here and many others could further fuel recent interest in the so-called exposome in humans (Nemkov et al., 2020b), with immediate translational relevance in fields such as transfusion medicine (e.g., lead in smoking blood donors, cadmium in coastal blood donors who regularly consume fish, etc.).

## Data Availability Statement

The original contributions presented in the study are included in the article/Supplementary Material, further inquiries can be directed to the corresponding author/s.

## Ethics Statement

The studies involving human participants were reviewed and approved by Canadian Blood Services. The patients/participants provided their written informed consent to participate in this study.

## Author Contributions

DS performed ICP-MS analyses. TN performed metabolomics analyses. SQ and WS performed stress experiments. DS and AD’A prepared figures. AD’A wrote the first version of the manuscript. All authors contributed to its finalization.

## Author Disclaimer

The views expressed herein do not necessarily represent the view of the Federal Government of Canada.

## Conflict of Interest

The authors declare that the research was conducted in the absence of any commercial or financial relationships that could be construed as a potential conflict of interest.

## Publisher’s Note

All claims expressed in this article are solely those of the authors and do not necessarily represent those of their affiliated organizations, or those of the publisher, the editors and the reviewers. Any product that may be evaluated in this article, or claim that may be made by its manufacturer, is not guaranteed or endorsed by the publisher.

## Footnotes

1
www.metaboanalyst.com
